# Dandy–Walker malformation: An incidental finding

**DOI:** 10.4103/0971-6866.64936

**Published:** 2010

**Authors:** Jyothi Tadakamadla, Santhosh Kumar, G. P. Mamatha

**Affiliations:** Department of Oral Medicine and Radiology, Darshan Dental College and Hospital, Udaipur-313 001, Rajasthan, India; 1 Department of Preventive and Community Dentistry, Darshan Dental College and Hospital, Udaipur-313001, Rajasthan, India

**Keywords:** Dandy–Walker, high arch palate, hypertelorism

## Abstract

Dandy–Walker malformation (DWM) is a rare intracranial congenital abnormality that affects the cerebellum and some of its components; particularly cerebellar vermis, fourth ventricle and is characterized by an enlarged posterior fossa. Although there is an extensive list of signs attributed to DWM, final diagnosis is solely dependent on imaging techniques as there are no signs that are characteristic of DWM. This article reports a case with DWM who was diagnosed by magnetic resonance imaging.

## Introduction

Dandy–Walker malformation is a rare congenital abnormality that affects the cerebellum and some of its components; particularly hypoplasia of cerebellar vermis, a cystic dilatation of fourth ventricle and is characterized by an enlarged posterior fossa.[1]

Dandy–Walker malformation was originally described in 1887 by Sutton and further characterized by Dandy and Black fan in 1914 followed by Tagart and Walker in 1942. Benda finally labeled this disease as dandy walker in 1954.[2]

Dandy–Walker complex has several variants, Dandy–Walker malformation (DWM) encompasses cystic dilatation of the fourth ventricle, complete or partial agenesis of cerebella vermis and enlarged posterior fossa while Dandy–Walker variant (DWV) comprises cystic posterior mass with variable hypoplasia of the cerebella vermis and no enlargement of the posterior fossa. However, the third variant mega-cisterna magna comprises enlarged cistern magna with normal cerebellar vermis and fourth ventricle.[3]

Infants with DWM may present with early signs such as vomiting, sleepiness, irritability, convulsions, unsteadiness and lack of muscle coordination.[4]

The clinical manifestations include psychomotor and growth retardation, hypotonia, strabismus, myopia, a short neck, microcephaly, brachycephaly, hypertelorism, antimongoloid slant of palpebral fissures, globulus large nose, large mouth with down turned corners, poorly lobulated ears, high arch palate, cleft palate, small hands and feet, clinodactyly, and the brachymesophalangy of the little fingers.[5]

Although it is said that clinical examination cannot replace any imaging modalities, DWM is such a condition that require imaging modalities to diagnose the disorder. Even though there are many signs, none of these are characteristic to diagnose individuals as DWM and diagnosis is solely based on imaging techniques. The present manuscript reports a case encountered in our clinic which was revealed as Dandy–Walker malformation by MRI.

## Case Report

A female patient of age 32 years was brought to the department of oral medicine and radiology for regular dental check up. She had a history of cranial trauma when she was three years and got unconscious for an hour. Her mother did not provide any history of medication during pregnancy. She was born at term and was the second child of non-consanguineous parents and the only affected case in the family.

**Figure 1:**
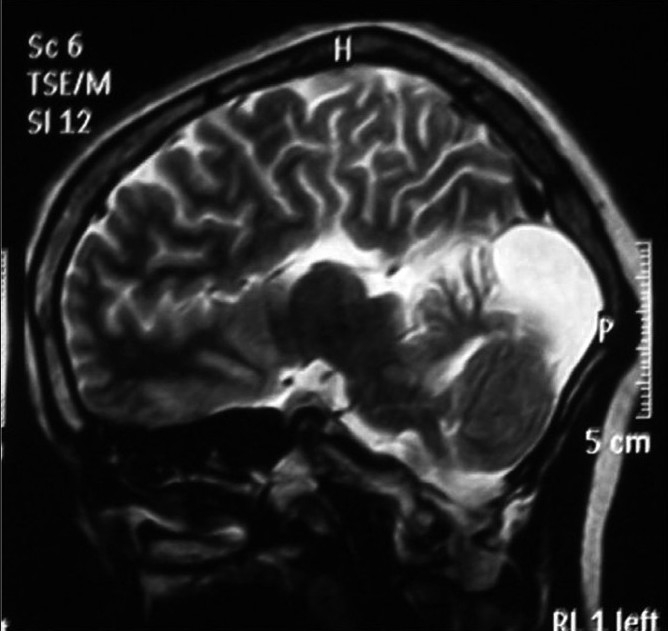
T2-weighed image depicting agenesis of the corpus callosum and colpocephaly.

**Figure 2:**
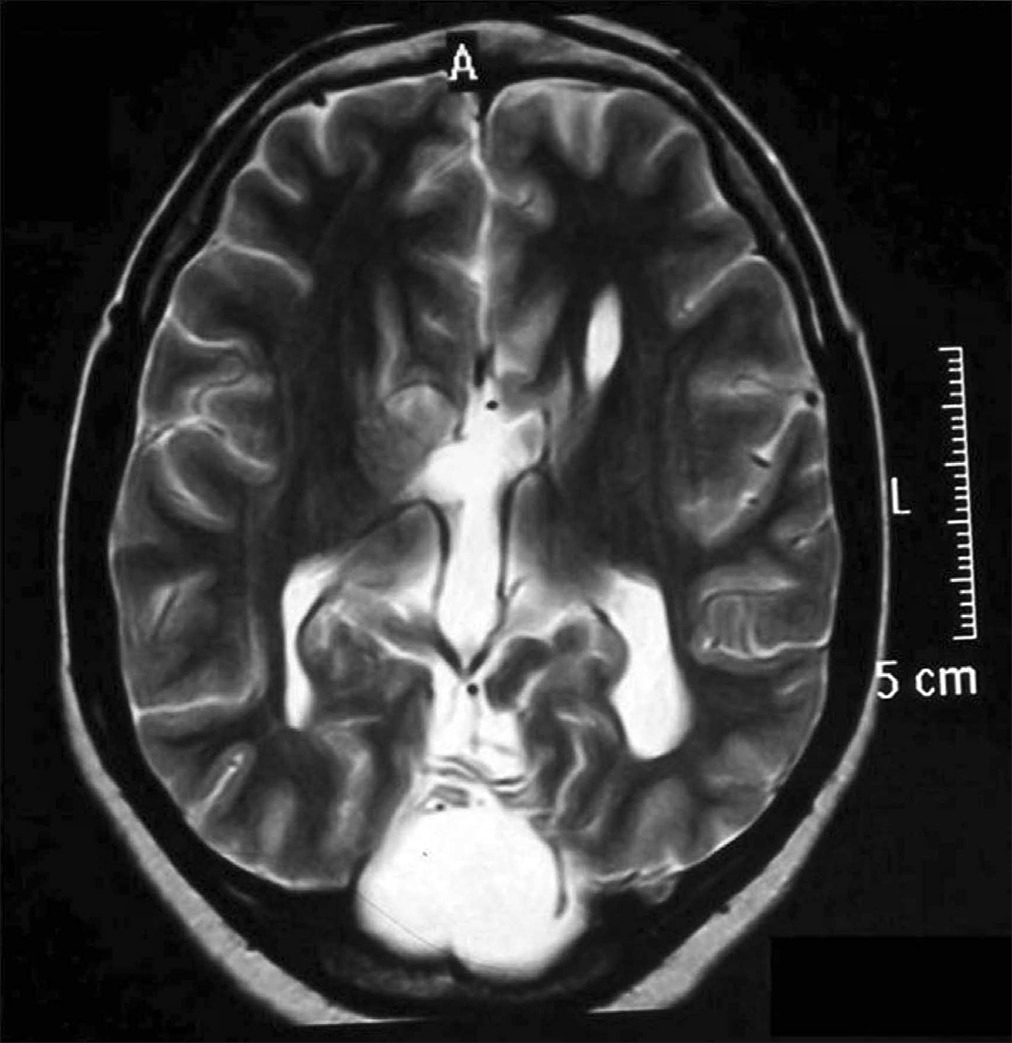
Well-defined axial cystic lesion in the posterior fossa in the midline communicating with the fourth ventricle in axial T2-weighed image.

**Figure 3:**
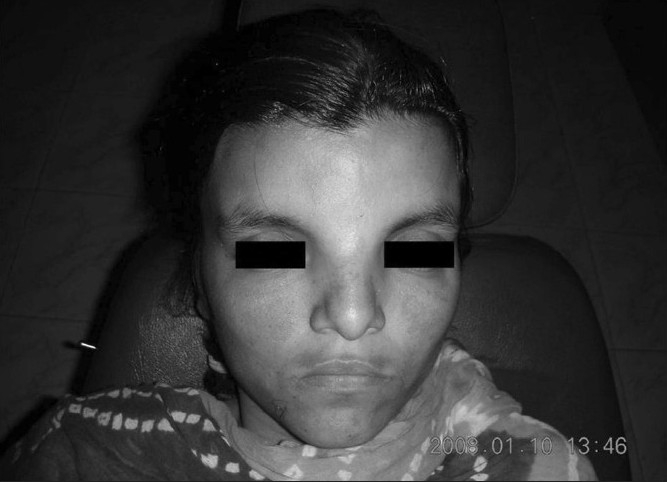
Facial photograph of the subject depicting frontal bossing with hyperterolism.

Personal history revealed irritability and drowsiness. Physical examination did not disclose any abnormalities but delayed milestones were reported. Her gait and all sensory examinations were normal while she had a low intelligence quotient (55). On extraoral examination, frontal bossing with hyperterolism [Figure 1] along with macrocephaly were observed. Furthermore, intra oral examination revealed high arch palate, poor oral hygiene with 47 and 48 being carious and Angle’s class 1 molar relation. Conventional radiographs revealed wide table with granular appearance of the skull. MRI of the brain was performed using T1, T2, flair and diffusion weighted sequences in multiple planes. Study of the MRI revealed a well-defined extra axial cystic lesion in the posterior fossa in the midline communicating with the fourth ventricle. There was an associated mild hypoplasia of the cerebellar vermis and the cerebral hemispheres were anterolaterally pushed toward the tentorial margins. The cystic lesion was causing mild indentation on the inner table of occipital horn. However, the posterior fossa was mildly enlarged in size [Figures 2 and 3]. These findings thus paved to the diagnosis of Dandy–Walker Malformation. Intra oral therapeutic procedures comprised oral prophylaxis and restorations.

## Discussion

DWM occurs as an autosomal dominant inherited disorder and occur in one in 25000 – 35000 pregnancies.[2] The syndrome can appear dramatically or develop unnoticed.

The gene locus for DWM is 3q24[6] and the presence of multiple congenital defects associated with DWM may shorten life span.[6]

Cases of Dandy–Walker malformation were reported more in females.[7] Various predisposing factors were reported such as infections, cranial trauma, chronic disturbance in cerebrospinal fluid pressure, persistence of embryonic tissue, vascular lesions, teratogens, rubella, alcohol and maternal diabetes.[2] In the present case, cranial trauma could have been the responsible factor.

Previous literature suggests that 40% individuals with DWM were normal intellectual while 40% had mental retardation and 20% were borderline.[2]

Cerebellum is believed to play a role in motor control, motor learning, and even cognition such as development of language and other cognitive skills.[8]

Thus, the effect of Dandy–Walker syndrome on intellectual development is variable, with some children having normal cognition and others never achieving normal intellectual development.

The present case had hyperterolism and high arch palate in accordance with a previous case.[5] Moreover, she was very drowsy and exhibited irritability, nausea sensation in accordance with the previous reports.[4]

Paladini and Volpe[9] in 2006 demonstrated that the degree of vermian hypoplasia correlates significantly with the occurrence and severity of mental retardation. Thus, it seems that the more abnormal the vermis is, the more poor the prognosis will be and our case had only mild hypoplasia of cerebellar vermis thus showing milder presentation.

Syndromes associated with DWM include “PHACE syndrome” (posterior fossa brain malformations, hemangiomas, arterial anomalies, coarctation of aorta and cardiac defects and eye abnormalities)[10] and Ellis-van Creveld syndrome.[11]

Through the use of modern diagnostic tools (e.g., ultrasound, CT, MRI, etc.), DWM is typically diagnosed in individuals before one year of age in approximately 76 to 80% of the cases. Historically, DWM was only found incidentally or by autopsy.[12] Today there are a number of different neuroimaging techniques that are used to diagnose and confirm diagnoses in fetuses, infants, and all others who may be suspected of having DWS. Adult cases of DWM for instance are usually diagnosed incidentally by neuroimaging methods or after minor head trauma as diagnosed in the present case.[13]

## Conclusions

Dandy–Walker malformation is a condition that can be diagnosed by advanced imaging modalities which are expensive to use in developing countries. Extensive research is required to identify or diagnose the disease at the earliest. Moreover, special care has to be taken by oral physicians for such medically compromised patients as they are unable to maintain their basic oral hygiene.
